# The Etiology of Acute Endocarditis

**Published:** 1888-03

**Authors:** 


### *THE ETIOLOGY OF ACUTE ENDOCARDITIS.*

*Medical News*, October 1, 1887: We have already referred to the observations of Orth, Philipowicz, Prudden and others, which demonstrate the constant associations of micro-organisms with the lesions of ulcerative endocarditis, and the possibility of the artificial production of the disease in animals with or without previous laceration of the valves. A number of forms have been cultivated, chiefly cocci, but, in one or two cases, small bacilli, and in two instances in which the endocarditis complicated pneumonia, the same kind of organisms were found in the lung and on the heart-valves.

Weichselbaum has recently studied the question with great care, and has given his results in the *Centralblatt für Bacteriologie und Parasitenkunde*, Bd. ii., No. 8. Cultures were made from fourteen cases of ulcerative endocarditis as soon as possible after death; in some instances, within an hour or two. In twelve, microbes were present; in six cases, the streptococcus pyogenes; in three, the pneumonia diplococcus; and in three, less common but equally distinctive forms. In the two negative cases, the vegetations were old and calcified. The experimental work confirms the results of previous observers regarding the feasibility of inducing artificial endocarditis.

A much-debated point, upon which we need further observations, is whether, in the ordinary verrucose, endocarditis micro-organisms are present in the vegetations. Klebs, Koster and Ziegler regard this form as also bacteritic, but Osler, Orth and others have failed to demonstrate their presence. Recently, E. Frankel and Sänger in *Virchow's Archiv*, Bd. 108, have found organisms in nine out of fourteen cases, and have shown that the colonies may be so scanty that the culture method gives much more definite results than can be obtained by section and staining. In two instances, however, Weichselbaum could not find them, and suggests, in explanation, that the vegetation may have been old and the organisms dead. More extended observations are required before we can attribute every case of warty endocarditis to the irritation of microbes on the valves.

